# Effects of sera taken from women with recurrent spontaneous abortion on sperm motility and apoptosis

**Published:** 2011

**Authors:** Tahereh Talaei-khozani, Zahra Borzoei, Soghra Bahmanpour, Jaleh Zolghadr, Sedigheh Dehbashi, Hamid Reza Zareh

**Affiliations:** 1 Department of Anatomy, Shiraz University of Medical Sciences, Shiraz, Iran.; 2 Department of Obstetrics and Gynecology, Shiraz University of Medical Sciences, Shiraz, Iran.; 3 Department of Immunology, Shiraz University of Medical Sciences, Shiraz, Iran.

**Keywords:** *Unexplained recurrent spontaneous abortion*, *Sperm*, *Apoptosis*, *Spermmotility*

## Abstract

**Background: **Recurrent spontaneous abortion impacts almost 1% of couples. The sera from women with unexplained recurrent spontaneous abortion (URSA) have toxic effects on embryos that grow in the uterus. Therefore, the abnormal condition of the uterus may also affect sperm qualities.

**Objective:** The objectives of this study were to search if these sera could induce DNA denaturation in sperm nuclei and also it could reduce sperm motility.

**Materials and Methods:** Sera of 20 women with URSA history and sera from 20 women with at least two healthy children were added to the sperms samples from 20 healthy men for 2 hours. The sperm motility was assessed after incubation with sera. The samples were stained with Tdt mediated dUTP nick end labeling (TUNEL) assay for DNA fragmentation. The samples were analyzed with flow cytometry and the percentage of the TUNEL positive sperms were calculated. The data were analyzed by t-test.

**Results:** The incubation of the sperm samples in sera with URSA lead to a decrease in the percentage of the motile sperm from 55% in control to 41% in the treated group, significantly (p=0.038). The percentage of the sperm with abnormal fragmented DNA increased after incubation with URSA (26.6%) compare to the control (21.2%); however, it was not significant.

**Conclusion:** It seems that sera from URSA patients could not induce a significant increase in the percentage of the sperms with nuclei contain DNA fragmentation. However, the sera of women with URSA could affect the fertility rate by reduction of the sperm motility.

## Introduction

Recurrent spontaneous abortion (RSA) is defined as three or more consecutive losses of pregnancy (1). Chromosomal (6%), anatomical (1%), hormonal (5%) and immunological (65%) factors are the etiology of RSA. The etiology of 23% of them is still unknown (2). 

Male factor was also considered as the etiology of RSA. Sperm motility, viability and function could lead to RSA (3). Reduction in the sperm function in couples with idiopathic RSA may lead to formation of unsustainable embryo and result in early pregnancy loss (4). It has been shown that sperm with head abnormalities, defects of chromatin condensation and irregular nuclei with vacuoles are likely to be as possible male factors that contribute to early pregnancy loss (5). The investigation revealed that male factors like FSH level, Johnson score, sperm status and motility affect the intracytoplasmic sperm injection outcome in azoospermia (6). 

The correlation between sperm DNA damage and pregnancy loss is controversial (7). However, it has been revealed that sperm with damaged DNA can fertilize the oocyte at IVF successfully (8) and has no impact on normal embryo development (9). There is some evidence that sperm DNA fragmentation is related to the risk of cancer development and reduction of longevity in the offspring (10, 11). Besides, the insignificant increase in spontaneous abortion rate was observed in patients with DNA fragmented sperm (12). Therefore, the integrity of the sperm DNA is important for having healthy offspring.

In a normal mating or during the performance of the gamete intrafallopian transfer and intrauterine insemination procedures, sperms are exposed to the uterine environment. Changes in the uterine condition may affect the sperm quality. Unexplained RSA may be attributed to modification of maternal serum composition. Investigations have revealed that sera from women with recurrent miscarriages or other reproductive failures were toxic to the preimplantation embryos (13, 14). The sera from women with RSA could also change the surface molecules like glycoconjugates (14). However, the mechanism for the action of sera embryo toxicity is unknown. 

The presence of maternal anti-paternal antibodies was shown in women with a history of recurrent abortions; however, it had no role in such abortion (15). Sperm antibodies from female sera inhibit IVF in humans (16). The presence of anti-FSH antibody against FSH of the semen in maternal sera (17) and the amount of sex hormones can also affect the sperm qualities. Sex hormone may impact the sperm motility (18) and also survival and apoptosis (19). Therefore, modification of such parameter in female sera may change the sperm qualities after copulation and lead to spontaneous abortion. Given the above considerations, the objectives of this study aimed to find the effects of sera from women with RSA on sperm DNA damage and sperm motility. 

## Materials and methods

**Patients:**

Blood samples were collected from 20 volunteer females with at least three or more successive miscarriages without any stillbirths. The project was approved by ethics committee of Shiraz University of Medical Sciences. The patients and their husbands had normal karyotype. They had no anatomical abnormalities. They also did not suffer from any systematic diseases like heart failure, agglutination disorders, lupus, and chronic renal failure. They also did not suffer from ovarian polycystic and endometriosis. Their endometrial biopsies were normal in the leuteal phase. The cause of abortion was diagnosed as unexplained recurrent abortion. The patients did not take any medication at the time of blood collections. As the control group, the sera were taken from 20 normal females volunteers. They did not experience any abortion and had at least two live born children. They were healthy and did not take any medication at the time of blood collection. 

**Serum preparation:**

The blood samples from the experimental groups (RSA patient) and control groups (normal females) were centrifuged for 10 min at 3000 rpm. All the sera were heat inactivated in 56ºC for 30 min and stored at -20ºC until used. 

**Semen samples preparation:**

The sperm samples were taken from 20 healthy males who visited the cytogenetic center for genetic consultation. Their semen samples were assessed as normal according to the WHO criteria (20). The liquefied samples were washed twice by Ham's F10. The culture medium was added to the pellets and divided into two parts; one part was considered as experimental and the other as control. Each part was centrifuged and the supernatant was discarded. 100µl of the culture medium containing 20% (21) sera from women with RSA history (experimental) and the same amount of sera from normal women (control) were added to the sperm palates, resuspended and incubated for 2 hours at 37ºC. The motility of the sperms was assessed according to WHO criteria (20) after incubation in the experimental and control groups' sera. The percentage of straight forward motile sperms was calculated. 

**Tunel assay:**

The TUNEL assay was conducted using an in situ cell death detection kit, fluorecein (Roche with cat. No. 11684795910). The incubated samples were centrifuged at 3000 rpm for 5 min to remove the culture media containing sera. About 2×10^6^ cells/ ml were washed with PBS three times. The sperm samples were fixed with 2% paraformaldehyde for 60 min at room temperature. The fixed samples were centrifuged and resuspended in PBS. The washed samples were permeablized in 0.1% Triton X-100 in 0.1% sodium citrate for 2 min on ice. Sperm samples were washed with 200 µl of PBS twice. The sperms resuspended in TUNEL reaction mixture. The mixture contained the enzyme solution (terminal deoxynucleotidyl transferase) and label solution (FITC labeled nucleotide mixture). For negative control, just FITC labeled nucleotide was added. The samples were incubated for 60 min at 37ºC in humidified atmosphere in dark. They were washed twice in PBS and the FITC labeled sperms were measured in the FL1 channel of flow cytometry. The percentages of TUNEL positive cells were analyzed by histogram using the WINmdi 2.7 software. The samples were also observed with florescence microscope. 

**Statistical analysis**

The comparison of the percentage of the TUNEL positive sperms and also the percentage of motile sperms between the experimental and control samples were analyzed by independent samples test, using SPSS version 11.5 for windows. p-value less than 0.05 was considered as significant. 

## Results

All semen samples were assessed as normal before the beginning of the experiment. The percentage of the motile sperm, morphology, count and also pH of the semen samples included in the study were normal according to WHO criteria. After incubation for 2 hours in the sera from women suffering from RSA, the sperm motility was decreased significantly as compared to the controls. In the semen samples exposed to the sera taken from RSA patients, the mean of the percentages of the straightforward motile sperms was 41%±4.1% while, the mean of the percentages of straightforward motile spermatozoa was 55%±3.4% in control samples (p=0.038). 

**Figure 1:**
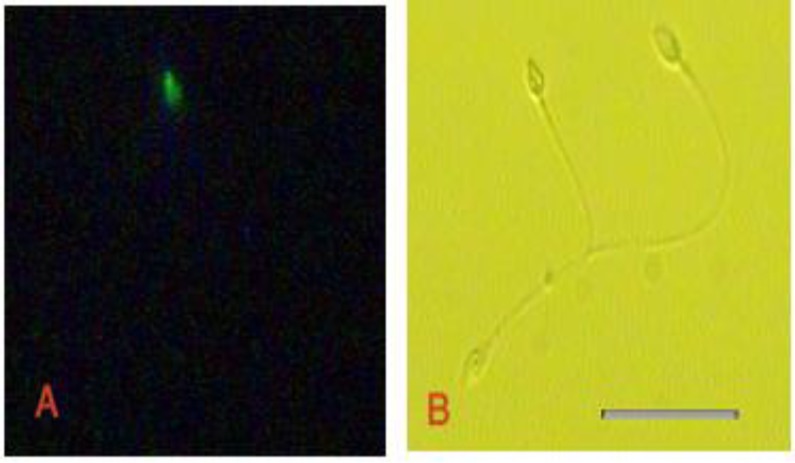
TUNEL positive sperm stained green (A), the sperms that did not react with TUNEL assay can be observed in phase contrast microscopy from the same region, (B). Scale bar is 20µm

**Figure2:**
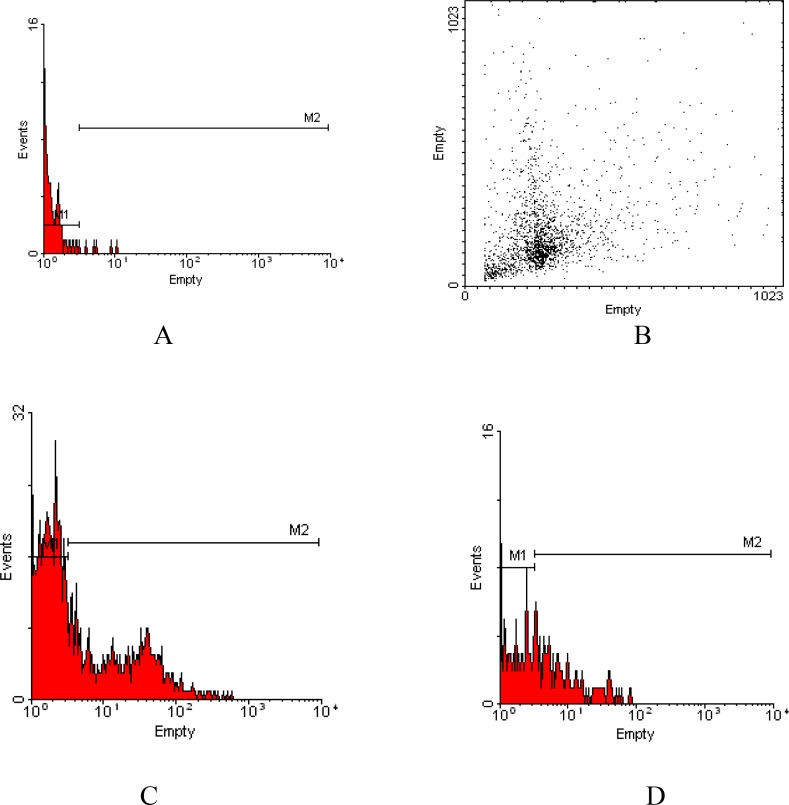
Comparison of the TUNEL positive sperms after exposure to the sera. Negative control (A), Dot plot (B), one sample that was exposed to sera from woman with RSA with 33.18% of TUNEL positive cells (C), one sample that was exposed to the sera from normal woman with 4.74% of TUNEL positive cells (D).

The TUNEL positive sperm head with green fluorescence is shown in figure 1. Flow cytometery assay showed the median value of the percentage of the TUNEL positive sperms was reduced after exposure to the sera from women with RSA; however, it was not statistically significant (p=0.722). In the semen samples exposed to sera taken from RSA patients, the median value of the percentage of TUNEL positive spermatozoa was 26.6%. The median value of the percentage of TUNEL-positive spermatozoa was 21.2% in control samples. Figure 2 demonstrates the histogram of the TUNEL assay analyzed by flow cytometry. The experimental semen contained less TUNEL positive cells that indicated sperms with DNA fragmentation nucleus. 

## Discussion

When the sperm enters the female reproductive tracts, it is faced with a microenvironment that can impact its quality. Follicular fluid constitutions are the main sperm microenvironment. Sera from women can be considered as an index of follicular fluid content, because many of the body fluids are derivatives of the serum. Sera and also follicular fluid contain many factors that may affect the sperm quality. The presence of the anti sperm-antibody (16), anti-FSH antibody in the maternal sera (17), cytokines (22) and the level of sex hormones in the sera can influence the sperm quality such as sperm motility. 

Our data indicated that the motility of the sperms exposed to the sera from women with RSA history decreased significantly as compared to the controls. It has been shown that cytokines, presence in the sperm microenvironment, may influence human sperm motility (23). The presence of oxidative stress agents is considered as another cause of pregnancy loss (24). Such an agent in the sera may decline sperm motility (25). Different amount of sex hormones in the sera may impact the sperm motility (18). Therefore, there are different factors in the sera of women with RSA that can influence sperm motility in different ways. 

There is some evidence indicating that apoptotic sperms can be motile (26). As our data indicated, the sera from women with spontaneous abortion could lead to a decline in the sperm motility, but it had no significant effect on the induction of apoptosis. Significant correlation was reported between sperm DNA fragmentation and sperm motility (27). The majority of the DNA damage encountered in human spermatozoa, caused by oxidative stress (28). There are also some reports that revealed correlation between oxidative stress agents in the sera and early pregnancy failure (29). This cytotoxicity might have also induced apoptosis in sperms as well. However, our data revealed that the sera were not toxic to sperms.

TUNEL positive sperm can be considered as apoptoic sperm (30). Apoptosis in ejaculated spermatozoa is a common phenomenon (31). Apoptosis was also observed in subfertile men (31, 32). Shen *et al* (2002) and Oosterhus and Vermes (2004) found that the median value of the sperms from subfertile men showed DNA fragmentation. The values were 15% and 20%, respectively. Piasecka *et al* (2007) also performed TUNEL assay in the semen samples. They found a median value of 11.69% of TUNEL positive cells in the semen with normal motile sperms. However, our data indicated that apoptotic sperms (21.2%) in normal semen samples were the same as those observed in subfertile men, after 2 hours of exposure to normal serum. Therefore, more sperms with DNA fragmented nuclei may be related to the serum exposure. 

The sera from women suffering from RSA increased the median value of the percentage of the apoptotic sperms; however, it was not significant. Depending on the cases, these sera may contain various factors like antisperm antibody (16) and anti FSH antibody (17) that could not induce apoptosis in a large scale during the sperms' journey in the uterus. 

Women experiencing recurrent spontaneous abortion have a higher frequency of infertility than the general population (33). The embryotoxic factors in the sera may persist cumulatively in the maternal system as a function of the cycles of spontaneous abortion (33). Therefore, as the number of abortion increases, the factors in the sera that influence sperm motility may lead to a decrease in fertilization rate. This may potentially cause secondary infertility. 

In conclusion, the sera from women with RSA can decline the sperm motility but they can not induce apoptosis significantly; however, the semen samples exposed to the sera led to an increase in the median value of the percentage of the apoptotic sperms. The decrease in motility was not related to the induction of apoptosis and it might have led to a reduction in fertilization rate. 
